# Rifampin-resistant/multidrug-resistant Tuberculosis in Alberta, Canada: Epidemiology and treatment outcomes in a low-incidence setting

**DOI:** 10.1371/journal.pone.0246993

**Published:** 2021-02-16

**Authors:** Brett D. Edwards, Jenny Edwards, Ryan Cooper, Dennis Kunimoto, Ranjani Somayaji, Dina Fisher

**Affiliations:** 1 Department of Medicine, University of Calgary, Calgary, Alberta, Canada; 2 Pharmacy Services, Alberta Health Services, Calgary, Alberta, Canada; 3 Division of Infectious Diseases, Department of Medicine, University of Alberta, Edmonton, Alberta, Canada; 4 Department of Microbiology, Immunology, and Infectious Diseases, University of Calgary, Calgary, Alberta, Canada; 5 Department of Community Health Sciences, University of Calgary, Calgary, Alberta, Canada; Chinese Academy of Medical Sciences and Peking Union Medical College, CHINA

## Abstract

Treatment of rifampin-monoresistant/multidrug-resistant *Tuberculosis* (RR/MDR-TB) requires long treatment courses, complicated by frequent adverse events and low success rates. Incidence of RR/MDR-TB in Canada is low and treatment practices are variable due to the infrequent experience and challenges with drug access. We undertook a retrospective cohort study of all RR/MDR-TB cases in Alberta, Canada from 2007–2017 to explore the epidemiology and outcomes in our low incidence setting. We performed a descriptive analysis of the epidemiology, treatment regimens and associated outcomes, calculating differences in continuous and discrete variables using Student’s t and Chi-squared tests, respectively. We identified 24 patients with RR/MDR-TB. All patients were foreign-born with the median time to presentation after immigration being 3 years. Prior treatment was reported in 46%. Treatment was individualized. All patients achieved sputum culture conversion within two months of treatment initiation. The median treatment duration after culture conversion was 18 months (IQR: 15–19). The mean number of drugs utilized during the intensive phase was 4.3 (SD: 0.8) and during the continuation phase was 3.3 (SD: 0.9) and the mean adherence to medications was 95%. Six patients completed national guideline-concordant therapy, with many patients developing adverse events (79%). Treatment success (defined as *completion of prescribed therapy* or *cure*) was achieved in 23/24 patients and no acquired drug resistance or relapse was detected over 1.8 years of median follow-up. Many cases were captured upon immigration assessment, representing important prevention of community spread. Despite high rates of adverse events and short treatment compared to international guidelines, success in our cohort was very high at 96%. This is likely due to individualization of therapy, frequent use of medications with high effectiveness, intensive treatment support, and early sputum conversion seen in our cohort. There should be ongoing exploration of treatment shortening with well-tolerated, efficacious oral agents to help patients achieve treatment completion.

## Introduction

Despite the availability of curative antimicrobial therapy for decades, *Mycobacterium tuberculosis* (TB) remains the most common infectious cause of death worldwide [1]. Following the development of early anti-mycobacterials including streptomycin, isoniazid, and rifampin in the 1940s to 60s, resistance naturally followed [2]. Treatment of drug-resistant TB offers a particular challenge due to lengthening of the treatment duration and requirement for increased number of antimicrobials to effect cure. Multidrug-resistant TB (MDR-TB) refers to resistance to the two core drugs against TB, isoniazid and rifampin, which together have decades of clinical experience and proven efficacy. This combination mediates early and sustained bactericidal activity and suppression of mycobacterial growth reducing the risk of relapse [3, 4]. Patients diagnosed with MDR-TB have lower treatment success (60–75%) than their drug-sensitive comparators (90–95%) [5–8] due to the required use of antimicrobials with decreased effectiveness, reduced protection against relapse, and increased toxicity, which demands longer treatment courses [9, 10]. The World Health Organization (WHO) recommends treating rifampin-monoresistant TB (RR-TB) with the same regimens as MDR-TB, highlighting the perceived importance of rifampin in preventing relapse. Additionally, treatment for MDR-TB is often greater than ten-fold more costly than drug-susceptible disease [11]. Despite all these challenges, recent developments have materialized in TB care with the release of two novel agents for TB (bedaquiline and delamanid) for the first time in decades [12], along with the repurposing of other antibacterial agents. Along with better data offering insight into the relative contributions of medications to successful treatment [6, 13], this has mounted a shifted interest internationally towards shortening treatment durations and using all oral regimens, although this remains a challenge due to medication access issues in Canada [14].

Canada is a low TB-incident country with an estimated rate of 4.9 per 100,000 population in 2017. Alberta represents one of three provinces/territories in Canada where the incidence rates exceed the national average [15]. Worldwide, the proportion of drug-resistant TB appears to be increasing [1, 11], but resistance rates in Canada have remained stable and below the global mean for decades [16–18]. MDR-TB represents an estimated 1.4% of those with TB disease in Canada, however resistance to ethambutol or pyrazinamide is encountered in two thirds of those isolates, further complicating the treatment [16].

We have previously explored the incidence of isoniazid-resistant TB in our center and found it to be consistent with national rates [18] and have thus focused this study on RR/MDR-TB. There has been a paucity of detailed Canadian data on RR/MDR-TB treatment outcomes. The morbidity, mortality, and cost associated with drug-resistant TB and the increase in migration from countries with high rates of RR/MDR-TB necessitate a close review of current practices and outcomes to guide further programmatic changes and ensure outcomes match those in other low TB-incident countries. Based on surveillance data we know Alberta to be a low TB-incidence setting and therefore anticipated we would be limited to a descriptive analysis. The objective of this study was to outline the epidemiology and describe the treatment, adverse events, and outcomes of RR/MDR-TB patients in Alberta, Canada over one decade.

## Materials and methods

We conducted a retrospective cohort study of all patients diagnosed with RR/MDR-TB in the province of Alberta, Canada (January 2007 –December 2017). TB care is centralized in Alberta and thus all patients with confirmed TB are treated in one of three clinics in the province by TB physician specialists. The clinics are multidisciplinary (physician, nursing, pharmacist, social worker) with intended bimonthly follow-up for two years beyond treatment completion for patients with drug-resistant disease. Significant treatment support is available: directly observed therapy is mandated by the province, outreach nursing support is available for home visits and patients have access to either home parenteral or ambulatory clinic parenteral therapy programs [18].

Mycobacterial culture growth and analysis for Alberta is completed at the Provincial Laboratory for Public Health (ProvLab). All samples are incubated at 37°C on solid (Lowenstein Jensen) and liquid media, utilizing the BACTEC Mycobacterium Growth Indicator Tube (MGIT) 960^TM^ system (Becton, Dickinson and Company, Franklin Lakes, NJ). Drug susceptibilities were determined using the BACTEC system (BACTEC 460TB^TM^ until 2010 and BACTEC 960^TM^ from 2010 onwards). Once the isolate is determined to be multidrug-resistant, second-line drug testing is completed at ProvLab and verified with solid-media and molecular testing at the National Microbiology Laboratory, Public Health Agency of Canada, for amikacin, kanamycin, capreomycin, ethionamide, PAS, rifabutin, streptomycin, linezolid, and moxifloxacin.

All patient with TB isolated from any body site with rifampin-resistance confirmed on phenotypic drug-susceptibility testing between January 1, 2007 and December 31, 2017 was included. Culture positive samples were identified as *respiratory* (all respiratory sites, including pleura, intrathoracic lymph nodes, larynx, and nasopharyngeal) or *non-respiratory* (all other sites) consistent with Canadian surveillance reporting [15, 19].

Patients were identified using the Integrated Public Health Information System (iPHIS), the Public Health database shared by all three clinics, which houses information on patient demographics, treatment data, adverse events, microbiology, and physician progress notes. The date of incident culture sample demonstrating RR/MDR-TB indicated the year of study inclusion.

Information collected included demographics, HIV coinfection, adherence, sputa smear/culture status, medication adverse events, and treatment outcome. *Treatment completion* was defined as completing a prescribed multidrug regimen individualized based on drug susceptibilities for RR/MDR-TB at the recommendation of the clinic physician. *Cure* was only applicable to patients with confirmed pulmonary disease and defined similarly to *Treatment Completion* but with the added requirement of at least three negative monthly sputum cultures following culture conversion. Other outcomes including *treatment failure*, *death*, *lost to follow-up*, and *not evaluated*, used previously reported WHO definitions [20]. We undertook a descriptive analysis of treatments utilized. Patients were designated as being treated with *guideline-based treatment* if their regimen was recommended by the most up to date Canadian guidelines in that year (either the 6^th^ or 7^th^ edition of the Canadian Tuberculosis Standards) [9, 21]. These were supplemented by contemporary MDR-TB WHO guidelines for decisions on duration of therapy [22–25].

Patient demographics and treatment data were provided in descriptive form with differences in means and proportions calculated using Student’s t-test and Chi-squared test respectively, with two-tailed significance set at <0.05, using STATA 15.1 (College Station, TX). Incidence was calculated using the provincial mid-year census data. We anonymized patient data by removing all directly identifiable information and replacing it with a unique study identification number with a protected key only available to the data investigators. All dates (treatment, immigration, hospitalization) were adjusted by a randomly selected number to preserve the anonymity of participants. Consent was waived due to the retrospective nature of the study. Ethical approval was obtained through the Conjoint Health Research Ethics Board (REB18-1673) at the University of Calgary.

## Results

### Patients

We identified 24 cases of RR/MDR-TB from a total 1720 TB cases in Alberta (1.4%) over the 11-year study period. The mean incidence rate was 0.06/100,000 persons per year. Demographic characteristics are outlined in Table 1. The mean age was 38.7 years (SD: 15.6) and 50% were female. There were no pediatric (<15 years old) cases. Only one patient was co-infected with HIV and was identified and initiated on ARV within eight weeks of commencing TB treatment.

**Table 1:** Baseline demographics and clinical features.

Total Patients: 24	
Age, mean (SD)	38.7 (15.6)
Female sex, no. (%)	12 (50)
Current Smoker, no. (%)	3 (12.5)
HIV (+), no. (%)	1 (4.2)
Viral load suppressed, no. (%)	0 (0)
Diabetes, no. (%)	3 (12.5)
Prior Treatment, no. (%)	11 (45.8)
Years from Prior Treatment, median (IQR)	2 (0.8–3.5)
Referred by Immigration Canada, no. (%)	12 (50)
Diagnosed at Time of Immigration Assessment, no. (%)	7 (29.2)
Foreign Born, no. (%)	24 (100)
Country of Origin, no. (%)	
• Philippines	12 (50)
• India	4 (16.7)
• Vietnam	2 (8.3)
• Ethiopia	2 (8.3)
• China	1 (4.2)
• Congo	1 (4.2)
• Eritrea	1 (4.2)
• Russia	1 (4.2)
Years from Immigration to Diagnosis of TB, median (IQR)	3.0 (0.3–7.3)
Site of Disease, no. (%)	
• Respiratory	16 (66.7)
• Non-Respiratory	7 (29.2)
• Both	1 (4.2)
Radiographic Distribution of Pulmonary Disease, no. (%)	
• Nodular/fibrotic	10 (41.7)
• Cavitary	6 (25)
• None	8 (33.3)
Site of Non-Respiratory Disease, no. (%)	
• Peripheral Lymphadenitis	4 (16.7)
• Musculoskeletal	3 (12.5)
• GU	1 (4.2)
• GI	0 (0)
• Pericardial	0 (0)
• CNS	0 (0)
• Cutaneous	0 (0)
• Blood/Disseminated	0 (0)
Least invasive sample able to achieve diagnosis, no (%)	
• Sputum	16 (66.7)
• Other (tissue, aspirate, BAL, bone, etc.)	8 (33.3)
Sputum AFB status at baseline, no. (%)	
• Smear (+)	6 (25)
• Culture (+)	16 (66.7)

All patients were foreign-born, with half of the cohort (12 patients) originating from the Philippines. The median time to diagnosis after immigration was 3.0 years (IQR 0.3–7.3). Eight patients (33%) were diagnosed with disease in each of the following timeframes: within 1 year of immigration, within 1–5 years, and greater than 5 years. Twelve patients (50%) had been referred upon immigration to the clinic for chest radiographic abnormalities by Immigration, Refugees, and Citizenship Canada (IRCC) [26]. Seven of these twelve referred patients (29% overall) were found to have active TB on immigration assessment, usually shortly after arrival to Canada. There were no Canadian-born Indigenous or non-Indigenous patients identified.

Eleven patients (46%) reported previous anti-TB treatment, nine of whom received treatment prior to immigration to Canada. The median time since the previous treatment was 1 year (0.8–3.5). The median self-reported treatment duration of previous treatment was 8 months (IQR: 6–12) with three patients reporting therapy was via Directly Observed Therapy (DOT). Two patients were being retreated within the study period after initially receiving treatment for fully sensitive and isoniazid-resistant TB respectively, both with excellent reported compliance (≥94%) prior to immigration. Alcohol and drug use/abuse were unreliably reported in the medical record and could not be quantitated.

### Diagnosis and microbiology

Seventeen patients in our cohort (71%) had confirmed respiratory disease, with 6/24 (25%) of the cohort sputum smear positive. Six patients (25%) had cavitary disease. Specific locations of non-respiratory disease are outlined in Table 1, with peripheral lymphadenitis being the most common non-respiratory site of disease. Two thirds of the cohort (66.7%) were diagnosed from cultures of spontaneously expectorated or induced sputum samples.

There was one case of rifampin monoresistant-TB and the remaining 23 were found to have resistance to both isoniazid and rifampin (Table 2). Nearly 50% of isolates had resistance to each of ethambutol and pyrazinamide. We found no resistance to second-line injectables but two cases demonstrated fluoroquinolone resistance. There was no difference in the mean number of medications resistant to between treatment naïve and experienced patients (p = 0.57). There was no difference in number of medications resistant to between patients from the Philippines and others in the study (p = 0.14, data not shown). Five patients isolated non-tuberculous *Mycobacteria* species during their treatment course.

**Table 2:** Resistance to antimycobacterials, by prior treatment status.

Drug	Prior Treatment for TB (n = 11) Proportion (%)	First Diagnosis of TB (n = 13) Proportion (%)
Isoniazid (>0.2 μg/mL)	10/11 (90.9)	13/13 (100)
Isoniazid (High Dose: >1 μg/mL)	7/8 (87.5)	4/5 (80)
Rifampin	11/11 (100)	13/13 (100)
Pyrazinamide	2/7 (28.6)	4/6 (66.7)
Ethambutol	6/11 (54.5)	5/13 (38.5)
Amikacin	0/7 (0)	0/8 (0)
Kanamycin	0/11 (0)	0/13 (0)
Capreomycin	0/11 (0)	0/13 (0)
Streptomycin	2/11 (18.2)	9/12 (75)
Ofloxacin	1/11 (9.1)	1/13 (7.7)
Moxifloxacin	1/7 (14.3)	1/8 (12.5)
Rifabutin	9/11 (81.8)	11/13 (84.6)
Ethionamide	5/11 (45.5)	7/11 (63.6)
Para-Aminosalicylic Acid	1/11 (9.1)	0/13 (0)
Linezolid	0/7 (0)	0/8 (0)
Total Drugs Resistant to, mean (SD)	4.5 (1.6)	4.9 (1.8)

Not all drugs listed had susceptibilities available for all isolates (indicated when numbers do not add up to total patient numbers).

Prior treatment status if reported by patient. BACTEC MGIT methodology used.

### Regimens

The majority of patients (92%) were treated in a large urban center (Calgary 12, Edmonton 10), and the remaining two rural patients received virtual physician assessment and care delivered locally by nurses. Treatment was individualized according to the drug-susceptibility profile of the isolate and patient comorbidity. There was significant variability in regimens initiated, both in content and in duration (Table 3). Amikacin was trialed in 83% of regimens, while fluoroquinolones (FQ) were utilized in 92% of cases, with the two exceptions being those with FQ resistance. Linezolid use increased during the study, utilized in 6/12 (50%) from 2007–2011 and 12/12 (100%) from 2012–2017, p<0.01. Similarly, clofazimine was used in 2/12 (16.7%) from 2007–2011 and 8/12 (66.7%) from 2012–2017, p = 0.02. The median total duration of therapy was 18 months (IQR: 14–20), with a median duration after culture conversion of 18 months [15–19]. During the intensive phase of treatment, the mean number of drugs utilized was 4.3 (SD: 0.8). During the continuation phase of therapy, the mean number of drugs utilized was 3.3 (SD: 0.9). All treatment was provided by directly observed therapy with mean adherence of 95% (SD: 5.9). Eighteen patients were initiated on a contemporary guideline-based regimen, however only six ultimately were able to complete one. Short duration of therapy was the most frequent reason for not completing a guideline-based regimen, occurring in 11/23 (48%) of patients. Treatment duration overall was short in many: 4/7 patients (57%) with extrapulmonary disease completed less than 18 months of therapy while 7/16 patients (44%) with pulmonary disease completed less than 18 months of therapy beyond confirmed culture conversion. However, treatment duration 12 and 15 months beyond culture conversion was achieved in 81% and 88% respectively. Three of the extrapulmonary patients had peripheral lymphadenitis as their sole disease site.

**Table 3:** Patient clinical course and outcomes (n = 24).

Patient, Year of Diagnosis	Site of Disease	Sputum Smear at Diagnosis	Prior Tx Year*	Daily Treatment Initiated, # of drugs**	# Hospital Admits, Duration	Duration of Treatment (months)^%^	Outcome	Follow-Up Duration (years)
30–35 y.o F (2008)	Pulmonary	+	1999	Ami-Lfx-E-Eto (4/2)	1, 4 days	18	Cure	6.8
30–35 y.o M^†^ (2008)	Lymph Node	-	-	Ami-Lfx-Lzd (3/2)	-	10	Treatment Completion	1.7
60–65 y.o M^$^ (2009)	Pulmonary	+	2008*	^$^H-Lfx-E-Z (4/3)	1, 16 days	17	Cure	1.6
35–40 y.o M (2010)	Pulmonary, Pleural	-	-	Ami-E-Mfx-Lzd (4/3)	3, 38 days	10	Cure	1.5
30–35 y.o M (2010)	Lymph Node	-	-	Ami-E-Lfx (3/2)	-	14	Treatment Completion	0.9
30–35 y.o M (2010)	Pulmonary	-	2008	Ami-Lfx-E-Z (4/3)	-	12	Treatment Completion	1.5
40–45 y.o M (2010)	Pulmonary	+	-	Ami-Mfx-Lzd-Eto-E (5/3)	1, 12 days	18	Cure	2
55–60 y.o M (2011)	Pulmonary	+	2006	Ami-Lfx-Eto-Z (4/3)	(uncertain)	17	Treatment Completion	1.1
30–35 y.o F (2011)	Mediastinal Lymph Node	-	-	Rfb-E-Z (3/3)	-	9	Treatment Completion	0
30–35 y.o F (2011)	Lymph Node	-	-	Ami-E-Mfx-Rfb (4/3)	-	16.5	Treatment Completion	1.8
35–40 y.o F (2011)	Pulmonary, Lymph Node	-	-	Lfx-Lzd-E-Z (4/4)	1, 9 days	20	Cure	2.1
25–30 y.o F (2011)	MSK	-	-	Ami-E-H(h)-Mfx-Lzd (5/3)	1, 8 days	24	Treatment Completion	0
20–25 y.o F (2012)	Pulmonary	-	-	Ami-E-Z-Lfx (4/4)	-	18	Cure	1.9
75–80 y.o F (2014)	Pulmonary	-	2013	Ami-Lfx-Z-Lzd (4/?)	-	-	Not Evaluated	Transfer
25–30 y.o M (2014)	MSK	-	-	Ami-E-Z-Lfx (4/4)	-	22	Treatment Completion	1.9
25–30 y.o M^&^ (2014)	Pulmonary	-	-	Ami-Lzd-Cs-Cfz-Bdq (5/4)	1, 19 days	17.5	Cure	2.2
30–35 y.o F (2014)	Genito-Urinary	-	2014*	Ami-Eto-Mfx-Lzd (4/3)	1, 12 days	13.5	Treatment Completion	2
45–50 y.o M^†^^&^ (2014)	Pulmonary	+	2013	Ami-Eto-Cfz-Cs-Lzd-Rfb (6/5)	1, 104 days	17	Cure	1.9
80–85 y.o M^†^ (2015)	MSK	-	2015*	Lfx-Lzd-E-Z (4/2)	3, 63 days	24	Treatment Completion	2.7
25–30 y.o M (2016)	Pulmonary	-	2014	Ami-Mfx-Lzd-E-Z (5/4)	1, 51 days	20	Cure	1.9
15–20 y.o F^†^ (2016)	Pulmonary	+	-	Ami-Lfx-Lzd-Cfz-Dlm (5/5)	1, 105 days	22	Cure	2.4
35–40 y.o F (2016)	Pulmonary	-	2015	Ami-Mfx-Lzd-Z-Bdq (5/4)	1, 26 days	18	Cure	1.2
35–40 y.o F (2016)	Pulmonary	-	2008	Ami-Lfx-Lzd-E-Z (5/3)	1, 21 days	18	Cure	1.5
25–30 y.o F (2017)	Pulmonary	-	-	Ami-Mfx-Lzd-E-Cfz (5/3)	1, 52 days	18	Cure	0.6

Ami = amikacin, H = Isoniazid, H(h) = high dose isoniazid (10 mg/kg), E = Ethambutol, Z = Pyrazinamide, Lfx = Levofloxacin, Mfx = Moxifloxacin, Eto = Ethionamide, Rfb = Rifabutin, CM = Capreomycin, CS = Cycloserine, PAS = Para Aminosalicylic Acid, Cfz = Clofazimine, Bdq = Bedaquiline, Dlm = Delamanid.

^†^Patient has a diagnosis of diabetes mellitus

^‡^Patient has a diagnosis of HIV.

*Daily Observed Therapy (DOT) utilized in Prior Treatment (as available, reported by patient or immigration documentation).

**(/) Number of Drugs in Intensive and Continuation Phases

^%^Treatment duration beyond culture conversion for pulmonary patients.

^$^Patient had rifampin-resistant TB

^&^Patient had MDR-TB with resistance to fluoroquinolones (“pre-XDR”).

### Outcomes

All patients demonstrated early and effective treatment response. Within the cohort, 22/24 patients (92%) were sputum culture-negative by one month of treatment. The other two were culture negative by the second month of treatment. There were no cases of culture reversion, with a mean number of post-treatment initiation sputum cultures (until 24 months) of 10.6.

Sixteen patients (67%) were hospitalized during the course of their treatment (Table 3). There were no deaths due to TB in this cohort.

Treatment success (*Completion* or *Cure*) was achieved in 96% of patients. There were no occurrences of failure, relapse, or resistance acquisition detected on post-treatment follow-up with median duration of follow-up of 1.8 years (IQR: 1.3–2.0) and all patients with pulmonary disease had end of treatment sputum collection to confirm smear and culture negativity. Two patients did not follow up after completing treatment.

Patient 9 had a very atypical treatment course. She was diagnosed based on intrathoracic lymphadenopathy without parenchymal disease. It was initially believed to be due to isoniazid-resistant disease and treated with rifampin, pyrazinamide, and ethambutol. However, 4.5 months into treatment, peripheral colonies on the sample were noted to show rifampin resistance. The patient was switched to rifabutin (confirmed susceptible), pyrazinamide, and levofloxacin and treated for another 4.5 months. She never represented to care.

Patient 8 underwent pneumonectomy. He had significant unilateral cavitary disease and previous treatment prior to immigration. Due to concerns of the risk of relapse, he completed 16 months of therapy prior to pneumonectomy with a subsequent 4 months post-operatively. He was followed for 13 months after treatment with no complications.

Patient 14 was designated as *Not Evaluated/Loss to Follow-up* as she returned to her country of origin after 6 months of therapy in Canada and we do not have outcome data for her.

Adverse effects were common and are outlined in Table 4. Nineteen patients (79%) required treatment modifications due to adverse effects, although three of these patients were successfully rechallenged with the discontinued medication. The majority of adverse events were Grade 1–2 and we are not aware of any adverse events requiring admission to hospital. Permanent hearing loss was not reported although follow-up audiology assessments were inconsistently documented. Non-resolving renal failure was not identified, even with frequent use of aminoglycosides. Permanent visual loss or neuropathy was also not reported. Cost of treatment could not be estimated.

**Table 4:** Medication adverse events.

Drug	Patients who used, no. (%)	Adverse Event(s), no. (%) *	Adverse Event(s) Description	Median Duration of Antimicrobial Use Prior to Discontinuation for Adverse Event; Months (IQR)
**Total Patients: 24**				
**Amikacin**	21 (87.5)	14 (66.7)	Ototoxicity: 10	3.8 (1.3–6)
			Intravenous catheter issues: 3	
			[Unable to determine]: 1	
**Bedaquiline**	5 (20.8)	2 (40.0)	Headache: 1	4.9 (4–5.8)
			Myositis: 1	
**Capreomycin**	2 (8.3)	0 (0)	-	-
**Clofazimine**	10 (41.7)	3 (30)	QTc Prolongation: 1	3.5 (2.7–7.9)
			Liver Injury: 1	
			Hyperpigmentation: 1	
**Cycloserine**	4 (16.7)	0 (0)	-	-
**Delamanid**	7 (29.2)	0 (0)	-	-
**Ethambutol**	18 (75.0)	3 (16.7)	Cutaneous: 2	4.6 (0.1–10.6)
			Visual symptoms: 1	
**Ethionamide**	7 (29.2)	5 (71.4)	GI: 2	1.8 (1.6–4.4)
			Cutaneous: 2	
			MSK: 1	
**Fluoroquinolone**				
•**Levofloxacin**	22 (91.7)	1 (4.5)	Liver Injury: 1	4.4
•**Moxifloxacin**	12 (50)	5 (41.7)	GI: 3, Tendinopathy: 1,	2.6 (1.3–6.8)
			QTc Prolongation: 1	
**Isoniazid (high dose)**	3 (12.5)	1 (33.3)	GI: 1	2.1
**Linezolid**	18 (75.0)	6 (33.3)	Hematologic: 5	7.1 (3.6–10.1)
			Peripheral Neuropathy: 1	
**Para-Aminosalicylic Acid**	4 (16.7)	3 (75.0)	GI: 3	0.5 (0.4–0.7)
**Pyrazinamide**	15 (62.5)	5 (33.3)	GI: 3	1.9 (0.6–3.8)
			Cutaneous: 2	
**Rifabutin**	5 (20.8)	0 (0)	-	-

*Those who took at least one dose.

## Discussion

The annual incidence of drug-resistant TB in Canada over the last decade has been 9% compared with 17% worldwide. The annual incidence of MDR-TB has remained stable in Canada at 1% of all isolates [16, 17], similar to our local findings. This incidence is significantly lower than the worldwide rates of RR/MDR-TB, which are estimated to range from 3.5% of new cases to 20% of retreatment [11]. The worldwide proportion of drug-resistant TB has been increasing for years for many reasons related to the difficulty in treatment [18]. Additionally, treatment of unrecognized isoniazid mono-resistance worldwide may be contributing substantially to the proliferation of MDR-TB [18, 27–30]. With worldwide travel and immigration continuing to increase, Canadian clinicians should expect to encounter more resistant isolates.

Our cohort with RR/MDR-TB was younger than that reported for drug-susceptible TB during the same time period [15], and this finding has been previously reported [11]. The lack of any cases in pediatric patients is consistent with a low incidence of both drug-susceptible and drug-resistant TB in this age group across Canada [16]. Prior treatment was frequently identified in our cohort and is a known risk factor for drug-resistant TB [8, 9]. Extensively drug-resistant-TB cases remain very uncommon in Canada [16, 31] and no cases were identified in our cohort nor reported in our province for over two decades [31].

Canadian surveillance data identified that between 2006 and 2016, 95% of all MDR-TB cases were in individuals born abroad, despite representing 20% of the population [32, 33]. This coincides with a shift in the patterns of immigration to Canada from low to higher incidence TB countries over the past several decades [8, 34]. Many of these countries also have high rates of MDR-TB and this emphasizes how drug-resistant TB incidence in Canada is inseparable from control of global TB resistance [8]. In our cohort, 100% of MDR-TB cases were diagnosed in foreign-born individuals, a median 3 years from time of immigration, with one-third presenting within 1 year. This is consistent with prior data reporting that cases of MDR-TB appear more likely to develop early in year 1 after immigration, perhaps suggesting recent acquisition in the home country and detection on immigration medical exam [8, 35–37]. It is likely to also represent some cases of relapse following recent treatment in the home country.

While half of our identified cases were in patients originally from the Philippines, 50% of these patients reported prior TB treatment (a recognized MDR-TB risk factor) and these accounted for 3% of all Alberta TB cases in individuals from the Philippines during the study period. Comparatively within the Philippines in 2017, RR/MDR-TB accounted for 2.6% and 28% of new and retreatment cases of TB, respectively [1]. Further, from 2006 through 2016, emigrants from the Philippines represented 24% of all immigrants to Alberta, the highest of any country [33]. Many of these individuals report prior work in a health-care setting, which is a known risk factor for TB infection. Therefore, this incidence is compatible with ethnocultural distribution within our province.

Half of our cohort were directed to our clinic for assessment upon immigration, while almost one-third were diagnosed with active disease at that time, due to referral from Immigration, Refugees, and Citizenship Canada (IRCC) [26]. It is uncommon for migrants to be diagnosed with active disease at the time of arrival [34]. More common is reactivation of disease in foreign-born over a subsequent year(s) as a complex interplay of host and environmental factors [34]. These cases represent important early ‘catches’, significantly lowering the time for, and subsequent risk of, transmission in the surrounding community [38].

MDR-TB treatment is limited by fewer drug options with lower effectiveness, higher toxicity, and prolonged treatment, all contributing to lower completion of therapy [9, 10]. Additionally, high rates of resistance to ethambutol and pyrazinamide, which both had nearly 50% resistance in our study, make empiric regimens very challenging. Variability of treatment regimens over the period of the study may be explained by changes in treatment guidelines and second-line drug availability [22, 24]. The benefit and tolerability of fluoroquinolones have been clearly demonstrated [13, 39–41] and these were utilized in all patients not precluded by resistance. Similarly, linezolid has been found to be an effective oral agent and clofazimine found to be both effective and well tolerated, and the use of both of these increased during the study [6, 13, 25, 41]. Following the introduction of bedaquiline in 2012 and delamanid in 2014, use of both increased in the second half of the study. Recent changes in the WHO recommendations for treatment of RR-TB has replaced injectables with bedaquiline in preferred RR/MDR-TB therapy [25]. This change has not yet been reflected in Canadian guidelines. In Canada, multiple internationally recommended medications for MDR-TB, including bedaquiline, clofazimine, and cycloserine, are not approved by Health Canada and require application for special access approval followed by subsequent release from the pharmaceutical company. This can introduce delay in administration of these drugs even if approval is granted. Also, the Health Canada Special Access Program [14] usually does not allow use of bedaquiline for solely extrapulmonary cases, which further reduces access to this drug.

Previous Canadian work demonstrated successful outcomes in 44% of patients [42]. Brode *et al* reviewed their cohort of 93 patients with MDR-TB and demonstrated much improved results with 84% of patients having successful outcomes [43]. Arguments put forward for such a dramatic increase in treatment success included a younger age, advent and use of later-generation fluoroquinolones [5], use of linezolid, and time effect allowing for improved MDR-TB care, including automatic inpatient admission for treatment initiation. Additional recent meta-analyses have typically found a pooled treatment success of 60–75% [5, 6]. Treatment was successful in 96% of our patients with no cases of delayed conversion, reversion, failure, relapse, or resistance acquisition. Successful treatment was thought related to individualized treatment regimens based on first and second-line drug susceptibility testing [8], universal use of DOT [44, 45], treatment support of a dedicated multi-disciplinary team, and close monitoring of side-effects.

Many of the regimens in the cohort had variability and brevity compared to concurrent Canadian guidelines. WHO guidelines at the time [22, 23] recommended a minimum of 18 months of therapy and Canadian guidelines deferred to even longer at 20–24 months [9, 21], however a recent systematic review and national guidelines have suggested that shorter durations may be just as effective for MDR-TB [46, 47]. Drug intolerance and toxicity contributed to the abbreviated length in many of the regimens. Additionally, a number of the cases were due to peripheral lymphadenitis, which represents localized pauci-bacillary disease that may not require prolonged therapy to prevent relapse. Early sputum conversion was identified for all of the patients in this cohort by two months and 93% with sputum culture positivity received 12 months of therapy post-sputa conversion, which may be a better indicator of successful treatment with low rates of relapse [48, 49]. The median number of medications used was 4 or more in the intensive phase and 3 or more in the continuation phase, which is believed to be the minimum number associated with treatment success [6, 25]. Additionally, the most up to date ATS/IDSA guidelines advocate that a minimum of 15 months beyond culture conversion may be sufficient for optimal treatment success of pulmonary disease [46]. This duration was achieved in 87% of cases. Most importantly, all patients reported a *successful* outcome and 17/24 (71%) were followed for a minimum of 18 months afterwards without evidence of relapse. Recent data shows that shorter treatment regimens are associated with higher treatment success and less loss-to-follow-up [50] and therefore the continued pursuit of shorter regimens that may keep patients engaged and free of cumulative toxicity is justified.

Individualization of treatment regimens is an important factor to consider in programmatic treatment strategies and has been highlighted in the recent North American guidelines [46]. Combining the routine availability of second- and third-line drug susceptibilities in our centre with knowledge of the relative effectiveness of medications [6, 13] contributes to predictors of success including early sputum conversion and limiting propagation of resistance. Similarly, individualization may prevent the onset of certain adverse events, as was done in our cohort for some who avoided amikacin due to baseline kidney dysfunction or bedaquiline due to baseline QTc prolongation. Finally, individualization of treatment regimens may improve patient engagement. For example, clinicians may rapidly address treatment intolerances by replacing the implicated agent with an effective alternative or perhaps negotiate treatment durations based on the site of infection, response to therapy, patient values, and estimated risk of relapse with medications utilized.

Adverse events were common, prompting treatment change in the majority of patients. Previous data has suggested a median of 29% of patients (IQR 16.1–53.3%) develop adverse events, with other reports reaching numbers similar to our findings of 79% [41, 51, 52]. The close clinical follow-up provided in our program may have led to increased detection of lower-grade adverse events, particularly gastrointestinal, and lower threshold for adjusting therapy. The observed high rates of ototoxicity with aminoglycosides are likely indicative of early discontinuation to avoid permanent damage. Unfortunately, audiology reports are not added to the patient electronic record for us to correlate. Nonetheless, this highlights the challenges of our currently available drug options and the ongoing need for new therapies.

The retrospective nature of our study introduces several limitations including the contextual rationale for some treatment decisions (why a drug was initiated/discontinued). This may also miscalculate adverse event frequency. Similarly, data on the severity of adverse events is limited due to inconsistent documentation of objective measures. The small sample size limits us to descriptive analysis with insufficient numbers for further statistical analysis, however we aimed to collect data that could be compiled with other patient-level studies [53]. Additionally, we sought to provide sufficient pragmatic program details for comparison to other low incidence countries. Successful treatment outcome definitions that were not intrinsically tied to durations of treatment may have increased our incidence of treatment success. However, evidence for treatment durations beyond culture conversion are evolving and may in fact allow shorter treatment durations that do not necessarily align with current international guidelines. Finally, while we cannot exclude the possibility of patients relapsing but presenting to care in another province or country, all patients with suspected or confirmed TB in Alberta receive care through our three clinics. Any suspicion of relapsed disease within the province would almost certainly be referred back to the TB program. The ProvLab is the only licensed facility to perform TB culture and liaises directly with the TB clinic. Death on treatment is reported in the provincial TB registry. Combined with the fact that patients willing to be followed are seen for two years following treatment completion–which is the most frequent timeframe for relapse—this reduces the likelihood of missed cases and would detect unexpected mortality.

## Conclusion

Our rates of RR/MDR-TB were consistent with Canadian rates over the same time period, with high incidence in foreign-born patients and with prior treatment. Patients were frequently detected at the time of immigration, which reduces the risk of community transmission. Individualized treatment regimens were variable, often shorter than contemporary Canadian guidelines and complicated by frequent adverse events. Despite this, treatment success was very high at 96%, likely attributable to significant treatment support, close monitoring, and the flexibility to adjust intolerable regimens while maintaining sufficient and effective medications. Patients in Canada would benefit from earlier access to new TB agents and all-oral regimens recommended by current international guidelines, along with ongoing exploration of shorter treatment duration to improve outcomes.
